# Habitat suitability and movement corridors of grey wolf (*Canis lupus*) in Northern Pakistan

**DOI:** 10.1371/journal.pone.0187027

**Published:** 2017-11-09

**Authors:** Muhammad Kabir, Shoaib Hameed, Hussain Ali, Luciano Bosso, Jaffar Ud Din, Richard Bischof, Steve Redpath, Muhammad Ali Nawaz

**Affiliations:** 1 Carnivore Conservation Lab, Department of Animal Sciences, Quaid-i-Azam University, Islamabad, Pakistan; 2 Wildlife Research Unit, Dipartimento di Agraria, Università degli Studi di Napoli Federico II, Via Universita n. 100, Portici, Napoli, Italy; 3 Institute of Biological Sciences, Faculty of Science, University of Malaya, Kuala Lumpur, Malaysia; 4 Snow Leopard Trust, Seattle, WA, United States of America; 5 Faculty of Environmental Sciences and Natural Resource Management, Norwegian University of Life Sciences, Ås, Norway; 6 School of Biological Sciences, University of Aberdeen, Scotland, United Kingdom; Sichuan University, CHINA

## Abstract

Habitat suitability models are useful to understand species distribution and to guide management and conservation strategies. The grey wolf (*Canis lupus*) has been extirpated from most of its historic range in Pakistan primarily due to its impact on livestock and livelihoods. We used non-invasive survey data from camera traps and genetic sampling to develop a habitat suitability model for *C*. *lupus* in northern Pakistan and to explore the extent of connectivity among populations. We detected suitable habitat of grey wolf using a maximum entropy approach (Maxent ver. 3.4.0) and identified suitable movement corridors using the Circuitscape 4.0 tool. Our model showed high levels of predictive performances, as seen from the values of area under curve (0.971±0.002) and true skill statistics (0.886±0.021). The main predictors for habitat suitability for *C*. *lupus* were distances to road, mean temperature of the wettest quarter and distance to river. The model predicted ca. 23,129 km^2^ of suitable areas for wolf in Pakistan, with much of suitable habitat in remote and inaccessible areas that appeared to be well connected through vulnerable movement corridors. These movement corridors suggest that potentially the wolf range can expand in Pakistan’s Northern Areas. However, managing protected areas with stringent restrictions is challenging in northern Pakistan, in part due to heavy dependence of people on natural resources. The habitat suitability map provided by this study can inform future management strategies by helping authorities to identify key conservation areas.

## Introduction

The distribution of species in space and time is a central topic in ecology. Species distribution models (SDMs) are increasingly important for investigating the requirements of species and for conservation planning [1–5]. Such models provide valuable quantitative information on the threats, such as areas of where there is high risk from humans, or where are the required resources [6] and they help identify conservation priorities [7–11].

The conservation of large carnivores remains challenging, in part due to a poor understanding of the complex spatial dynamics that facilitate population persistence [12]. The habitat requirements of such species deserve particularly close attention because they generally require large home ranges, are negatively impacted by changes in land use and are killed because of the threats they pose to livelihoods [13–16]. The grey wolf (*Canis lupus*) is a prime example of the drastic reduction in former ranges as a result of intensive persecution. Wolves were once widely distributed throughout the Palearctic and Nearctic biogeographic areas [17, 18]. However, global wolf range has decreased by 33% over the last century [18]. In many areas, agricultural expansion into marginal areas of wolf habitat has increased depredation of livestock and subsequently increased poaching, resulting in a numerical and spatial contraction of grey wolf populations [19].

A major concern of modern conservation efforts is identifying remaining habitat that is suitable for a species to occupy [19–23]. SDMs have proven to be effective at predicting habitat suitability for large carnivores, including wolves [24–26]. Habitat and conflict management can be implemented using the results from monitored wolf populations once potential areas are identified. In addition, knowledge of potentially suitable wolf habitat can be integrated into landscape planning [8]. Indeed, evidence from elsewhere suggests that map-based conservation planning can help facilitate human-wolf coexistence by identifying areas where the potential conflict caused by livestock depredation is high [27, 28].

Several studies have shown that the long-term survival of large vertebrates is achieved by both protecting source populations and providing dispersal opportunities between suitable patches [29,30]. Ecological corridors can help them connect local populations, allowing individuals free dispersal between populations [31]. Wolf dispersal patterns across the landscape can better predict where new wolf populations may appear [20]. Animals use a wide variety of mechanisms to select suitable habitat and being aware of habitat use details is important for corridor design [32]. Connectivity analysis is particularly important for wolves because allow them to know this animal can move through the existing habitat [33].

Wolf populations in Pakistan have suffered population declines and range contraction [34, 35]. They are now confined to remote, barren, mountainous regions and extensive deserts [36]. Numerous factors are thought to be responsible for their decline. The expansion of agricultural practices and land conversion has caused habitat loss. The movement of herders up the altitudinal gradient because of climate warming has further reduced available habitat and increased the impact of retaliatory killings of wolves. These predators move to lower altitudes during heavy snowfall, further increasing the chances of being killed due to livestock depredation [37].

Although a several studies have addressed GIS and modelling analyses of *C*. *lupus* in different area in the world [24–26], no research has been conducted on grey wolf in northern Pakistan. Our goal was to model, through the use of non-invasive survey data from camera traps and genetic sampling, a habitat suitability model for grey wolves in northern Pakistan and to explore the extent of connectivity between populations. We identified: a) the first geographical distribution analysis for *C*. *lupus* in northern Pakistan and which ecological factors may be limiting the species distribution in the study area; b) the corridors in northern Pakistan where the landscape would facilitate dispersal of *C*. *lupus* to provide an understanding of landscape permeability for large carnivores in a largely unsuitable matrix and to present conservation agencies with useful information should grey wolves continue to disperse into the region.

## Materials and methods

### Study area

**Fig 1:**
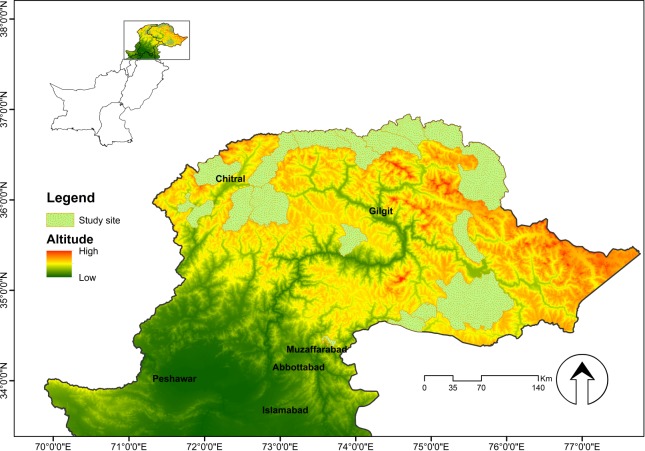
Study area (Northern Pakistan). Scales show the altitude ranging from low (dark grey) to high (red).

The Northern Areas of Pakistan (35-37^o^ N and 72-75^o^ E) are dominated by glaciated mountains crests, narrow valleys, ravines, cliffs and rough ridges [38]. They fall in the watersheds of the Karakorum, Himalaya and Hindu Kush mountain ranges (Fig 1). The Karakoram-Pamir landscape is a combination of several unique agro-ecological units that merge into one another [39]. Climatic conditions vary widely, ranging from the monsoon-influenced moist temperate zone in the western Himalayas to the semi-arid cold deserts of the northern Karakorum and Hindu Kush [40]. Four vegetation zones can be distinguished along the altitudinal gradients, namely alpine dry steppes, subalpine scrub zones, alpine meadows and permanent snowfields [38]. These varied climatic conditions and ecosystems support rare and endangered animals such as the snow leopard (*Uncia uncia*), brown bear (*Ursus arctos*), grey wolf (*Canis lupus*), Himalayan lynx (*Lynx lynx*), Marco Polo sheep (*Ovis ammon polii*), musk deer (*Moschus chrysogaster*), blue sheep (*Pseudois nayaur*), Himalayan Ibex (*Capra ibex sibirica*), flare-horned markhor (*C*. *f*. *cashmirensis*), Ladakh urial (*Ovis orientalis vignei*) and woolly flying squirrel (*Eupetaurus cinereus*).

### Genetic sample collection

The field surveys were conducted during the period 2010–2012 within the following protected areas (PAs): Machiara National Park, Musk Deer National Park, Khunjerab National Park, Broghil Valley National Park, Qurumber National Park, Shandoor-Handrab National Park, and Deosai National Park, as well as outside the PAs in Laspur, Yarkhun, Misgar, and Chapursan, covering suspected wolf range. We covered seven national parks and eight non-protected study sites. Survey areas were divided into grids cells of 5 × 5 km (except in Khunjerab National Park and Shimshal where the grid size was taken as 10 × 10 km) on GIS maps. Surveys points were randomly selected within each grid cell and a 50 m radius around each point was searched for wolf scats. We searched 1,736 points within the study area. We also searched for scats at camera trap locations and whilst hiking along livestock trails and manmade tracks (which were also used by wildlife). About 1,000 scat samples of carnivores were collected and preserved in 20 ml bottles filled with 95% alcohol.

We confirm that: 1) the institution leader of this research (Carnivore Conservation Lab, Department of Animal Sciences, Quaid-i-Azam University, Islamabad) has all field permit to work in all protected areas; 2) the owner of the land in non-protected study sites gave us the authorization to conduct the study on this site; and 3) no wolfs were harmed and sacrificed during this research. We only have collected wolf scats and image by camera traps.

### DNA analysis and species identification

Deoxyribonucleic acid (DNA) was extracted from 15 mg of feces using the DNeasy Blood and Tissue Kit (Qiagen, Hilden, Germany) recovered in a total volume of 200 μL. Blank extractions were systematically performed to monitor possible contaminations. Species identification was performed through next generation sequencings (NGS) by amplifying DNA extract using primer pair 12SV5F (5’-TAGAACAGGCTCCTCTAG-3’) and 12SV5R (5’- TTAGATACCCCACTATGC-3’) [41] targeting about 100-bp of the V5 loop of the mitochondrial 12S gene [42]. The sequencing was carried out on the Illumina Genome Analyzer IIx (Illumina Inc.), using the Paired-End Cluster Generation Kit V4 and the Sequencing Kit V4 (Illumina Inc.), following the manufacturer’s instructions. The sequence reads were analyzed using OBI Tools (http://www.prabi.grenoble.fr/trac/OBITools). Taxon assignation was achieved using the ecoTag program [43] in comparison with a reference database for vertebrates. This reference database was built by extracting the relevant part of the mitochondrial 12S gene from the European Molecular Biology Laboratory’s (EMBL) nucleotide library using the ecoPCR program [44]. Genetic results revealed identification of 80 samples belonging to wolves.

### Camera traps

Camera traps were installed in 798 locations during the period 2009–2017 and were separated by a horizontal buffer of at least 1 km (Table 1). Camera trap locations were identified based on landscapes characteristics—ridges, cliff bases, draw—preferred by carnivores and the presence of carnivore signs [38]. A single motion-triggered digital camera with infrared flash (HC500/PC900, Reconyx, Holmen, WI, USA) was deployed at each location on a steel pole (50–60 cm) driven into the ground. Camera traps were set to take consecutive images (1-s picture interval) when triggered and were typically kept active at a given location for 10–40 days [38]. Camera trap sites were baited with fish oil. Commercial trapping scent lures were deployed in some randomly selected sites [45].

**Table 1:** Details of camera trapping studies and photo captured record of wolf in Northern Pakistan.

N°	Site	Year	N° camera station	Camera station with wolf presence
1.	Chitral	2006	19	1
2.	Chitral	2007	18	5
3.	Chitral Gol National Park	2008	21	3
4.	Chitral Gol National Park	2009	20	0
5.	Tooshi game reserve	2009	30	0
6.	Khunjerab National Park	2010	10	0
7.	Laspur valley	2010	20	0
8.	Khunjerab National Park	2011	86	1
9.	Shimshal	2011	36	0
10.	CGNP, TGR & Buffer	2011	22	11
11.	Broghil & Qurumber	2012	80	6
12.	Deosai National Park	2013	116	9
13.	Yarkhun valley, Chitral	2013	58	0
14.	Misgar & Chapursan	2013	59	0
15.	Astore valley	2013	25	0
16.	Musk deer National Park	2014	36	1
17.	Khanbari, Diamer	2014	48	11
18.	Tirch valley, Chitral	2015	26	0
19.	Hisper valley, Nagar	2016	38	0
20.	Bhasha valley, CKNP	2017	30	3

### Model preparation: Selection of presence data and environmental variables

Records obtained by scats collection and camera trapping of *C*. *lupus* were screened in ArcGis (version 9.2) for spatial autocorrelation using average nearest neighbour analysis to remove spatially correlated data points and guarantee independence [46–48]. After this selection, from an initial dataset of ca. 131 presence records, only 25 unrelated locations were used to generate current SDMs of *C*. *lupus*.

To produce SDMs for *C*. *lupus* in northern Pakistan, we considered initially a set of 28 environmental variables. We included altitude, 19 bioclimatic variables, land cover, slope, soil type, distance to roads, distance to rivers, distance to settlements, vector ruggedness measure and normalized difference vegetation index. Bioclimatic variables and altitude were obtained from WorldClim database (www.worldclim.org/current) [49]. Land cover was obtained from the Global Land Cover 2000 (available from https://lta.cr.usgs.gov/glcc/globdoc2_0). Distance to roads, distance to rivers, distance to settlements were calculated by using Euclidean distance tool in Arc GIS 10.0. Soil (FAO, 2003, digital soil map of the world), vector ruggedness measure (SRTM 90m DEM by Center for Nature and Society, Peking University.) and normalized difference vegetation index were obtained from NASA website (http://modis-land.gsfc.nasa.gov/vi.html). The MODIS normalized difference vegetation index product is computed from atmospherically corrected bi-directional surface reflectance that have been masked for water, clouds, heavy aerosols and cloud shadows. Global MOD13A2 data are provided every 16 days at 1-kilometer spatial resolution as a gridded level-3 product in the Sinusoidal projection.

In order to remove any variables that were highly correlated before generating the models, we calculated a correlation matrix using Pearson’s technique and selected only the variables for which r < 0.70 [50]. From this first set of predictors, we selected only the variables that were most representative of the species’ ecological requirements [12, 16, 17, 18, 20, 24, 25, 26, 33, 34]. After this analysis, eight environmental variables were selected considering their applicability to the scale of our study area, relevant predictive power, and their suspected biological importance [51, 52]. All the variables were prepared—conforming cell size [30-arc second resolution (0.93 × 0.93 km = 0.86 km^2^ at the equator)], geographic extent, projection, and ASCII—using the ‘resample’, ‘clip’, ‘mask’, and ‘conversion’ tools in Arc GIS 10.0. Finally, the following eight environmental variables were used for model training: distance to roads (m), distance to rivers (m), mean temperature of wettest quarter (°C), mean diurnal range (°C), soil, annual precipitation (mm), altitude (m) and global land cover 2000.

### Maxent model

SDMs rely on presence-absence data or presence-only data [7, 9, 53–55]. The use of presence-only is recommended when absence data has a high degree of uncertainty relative to presence data, which is especially true when detection rates are poor [25,26]. We modeled wolf distribution using Maxent (ver. 3.4.0) as it is recognized as a better performer with presence-only data, especially with small numbers of occurrence points [56–58]. To build the models, we used the presence records (defined “sample” in Maxent) of *C*. *lupus* selected as described above and the environmental variables (defined “environmental layers” in Maxent). In the setting panel, we selected the following options: auto features; random seed; write plot data; remove duplicate presence records; give visual warming; show tooltips; regularization multiplier (fixed at 1); 10,000 maximum number of background points; 1,000 maximum iterations; and, finally, we achieved a 20 replicates effect with cross-validation run type as suggested by Pearson et al. [59] for testing small samples, this run type makes it possible to replicate n sample sets removing a locations at each step [47, 60, 61]. All other parameters were left by default. These settings are conservative enough to allow the algorithm to get close to convergence and optimize performance [62].

The final logistic output gave suitability values from 0 (unsuitable habitat) to 1 (suitable habitat). The 10 percentile training presence (i.e. the value above which the model classifies correctly 90% of the training locations) was selected as the threshold value for defining the species’ presence. This is a conservative value commonly adopted in species distribution modelling studies, particularly those relying on datasets collected over a long time by different observers and methods [11, 47]. This threshold was used to reclassify our model into binary presence/absence map.

We used Jackknife sensitivity analysis to estimate the actual contribution that each variable provided to the geographic distribution models. During this process, Maxent generated three models: first, each environmental variable was excluded in turn and a model was created with the remaining variables to check which one of the latter was the most informative. Second, a model was created by individually by adding each environmental variable to detect which variable had the most information not featuring in the other variables. Third, a final model was generated based on all variables. Response curves derived from univariate models were plotted to know how each environmental variable influence presence probability.

### Model validation

We tested the model with different validation methods: the receiver operated characteristics, analyzing the area under curve (AUC) [63] and the true skill statistic (TSS) [64].

AUC assesses the discrimination ability of the models and its value ranges from 0 (equaling random distribution) to 1 (perfect prediction). AUC values > 0.75 correspond to high discrimination performances [63]. TSS compares the number of correct forecasts, minus those attributable to random guessing, to that of a hypothetical set of perfect forecasts. It considers both omission and commission errors and success as a result of random guessing; its values range from -1 to +1, where +1 corresponds to perfect agreement and zero or less to a performance no better than random [64].

### Modelling potential movement corridors

A spatial corridor model was developed using the distribution map of wolves in Circuitscape 4.0 software (http://www.circuitscape.org/downloads) [65]. We used Circuitscape 4.0 to model connectivity and movement corridors of grey wolf in Pakistan across the landscape. Circuitscape treats the landscape as a conductance surface, where each pixel represents a resistor with an assigned resistance value. Pairwise electrical resistances between locations are calculated by running a theoretical electrical current between each population pair, with one population being set as the current source and the other as the ground [65]. Contrary to least cost resistance methods, Circuitscape does not assume that animals disperse according to previous knowledge of the surroundings, but is based on random walks [65]. It thus links populations through multiple pathways [65], such that connectivity between habitat patches increases according to the number of connected pathways, and the effective resistance between two populations is derived from the overall resistance across all pathways. We used SDM output as conductance layer and 24 nodes to run movement corridors of grey wolf in Circuitscape 4.0. The nodes were used to represent different areas where we have confirmed wolf presence in northern Pakistan. Clearly, we have not used all the nodes to run Circuitscape because otherwise this procedure would become too complex. We have used a very low number of nodes and have chosen them as the most important areas of wolf movement in northern Pakistan. We converted the nodes into a grid file in Arc GIS 10.0. Both the habitat suitability map (created by Maxent) and the nodes file were converted into ASCII format for a Circuitscape model run. We used the option of conductance instead of resistance because the landscape is represented as a conductive surface with low resistances assigned to landscape feature types [9, 66, 67]. Finally, the final map of movement corridors was reclassified into three categories, respectively, low, moderate and high to better represent the most important areas for *C*. *lupus* movements.

## Results

### Camera traps and genetic analysis

**Fig 2:**
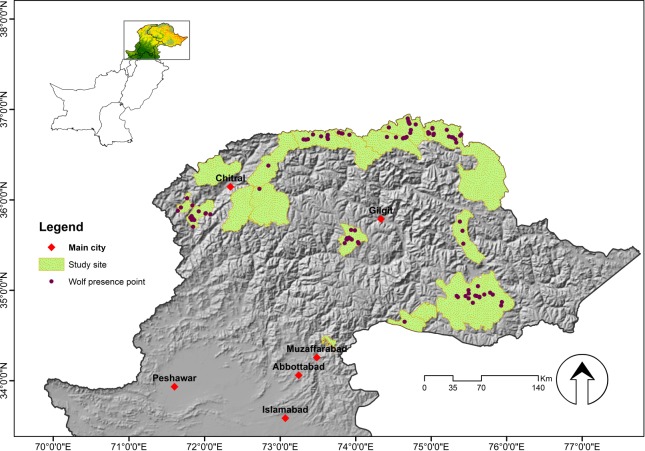
Genetic or photographic detections of wolves in the study area. The presence points were obtained from camera trap results and DNA analysis of scat samples collected from the Northern Areas of Pakistan (2009–2015). Altitude map in grey.

We obtained 51 wolf presence records from camera traps and 80 from the genetic analysis of scat samples collected from the distribution range of wolves (Fig 2). Most presence records were obtained from national parks. Presence records along the altitudinal gradients ranged from 3,000 m (Musk Deer National Park) to 4,700 m (Khunjerab National Park). PAs with higher frequency of presence records were Deosai National Park (Himalayan range), Chitral Gol National Park (Hindu Kush range), Khunjerab (Karakorum range) and Broghil National Park (Pamir range). Outside the PAs, the highest wolf encounter was recorded from the Khanbari Valley in Gilgit-Baltistan. There was no presence record from Terich, Astor, Misgar, Chipurson, Shimshal and Hisper Hooper Valley. Overall, wolf detection was low, suggesting thin and patchy population.

### Habitat suitability and model validation

**Fig 3:**
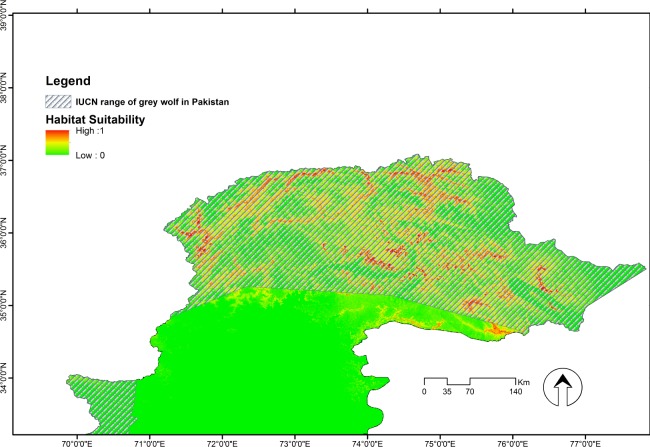
Wolf habitat suitability in Northern Pakistan, generated through Maxent. Scales show the probability of presence ranging from 0 to 1.

**Fig 4:**
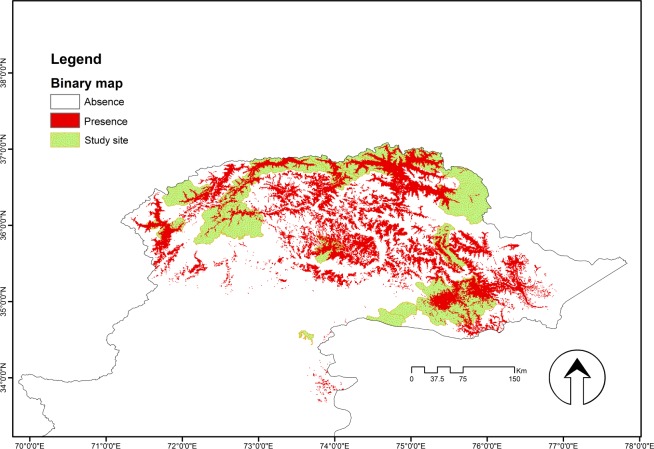
Binary map of *C*. *lupus* in Pakistan. White = Absence; Red = Presence.

The Maxent model suggested that there was suitable wolf habitat within the areas chosen as suspected wolf habitat range (Fig 3). The binary map discriminated between areas typically used by wolves and those considered unsuitable (Fig 4). The most suitable areas identified from the models were located predominantly within PAs and most inaccessible areas with minimum human disturbance, and overall, mainly along the narrow valley and around summer livestock pastures. The model suggested that there was less suitable habitat in lower altitude areas with more human access.

In relation to the distribution range, suitable areas were quantified based on habitat suitability modelling (Fig 4). The model predicted ca. 23,129 km^2^ of potential distribution of wolf in Northern Pakistan.

The jackknife-cross-evaluation test yielded the relative contribution and permutation of each environmental variable using Maxent. Distances to road, mean temperature of the wettest quarter and distance to river contributed most to the model. Soil, altitude, annual precipitation and land cover contributed relatively little. Response curves showed how the logistic prediction changed as environmental variables varied while keeping all other environmental variables at their average sample values. The probability of wolf presence was 0.7 at 0 km^2^ of distance from the road and stream, but it drastically to 0.05 at a distance of 0.5 km^2^. Mean temperature of the wettest quarter ranged from -10°C to 10°C with a max value (0.6) of probability of presence around 0°C.

**Fig 5:**
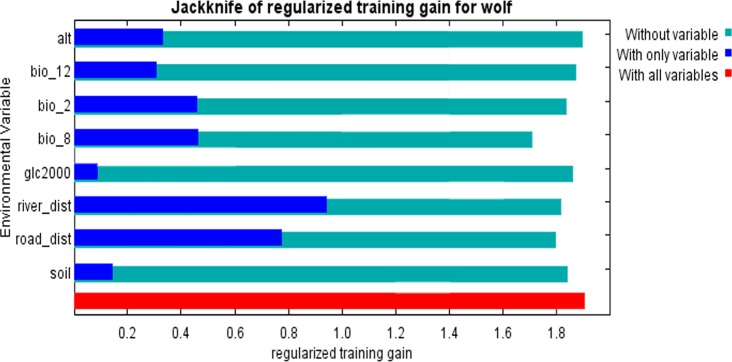
Jackknife analysis of variables. It shows how important each variable is in explaining wolf presence when used separately (cobalt blue), and how the model is affected when each variable is left out (aqua). Dark blue bars = Importance of single variable, light blue bars = loss in model gain, when variable is omitted. Red bar = total model gain. Alt = Altitude; bio_12 = Annual Precipitation; bio_2 = Mean Diurnal Range (Mean of monthly); bio_8 = Mean Temperature of Wettest Quarter; glc2000 = Global land cover 2000; road_dist = Distance to roads; river_dist = Distance to rivers; Soil = Soil.

The jackknife test also revealed the importance of different variables and their impact on model efficacy. Distance to river was the most important variable in determining model prediction in training, test and AUC evaluation. The distance to river increased the gain more than any other variables when added in isolation. On the other hand, the mean temperature of the wettest quarter variable decreased the gain most when omitted i.e. it contains the most information not present in other variables (Fig 5).

Finally, our model showed high levels of predictive performances as can be seen from the values of area under curve (0.971±0.002) and true skill statistics (0.886±0.021).

### Potential movement corridors

**Fig 6:**
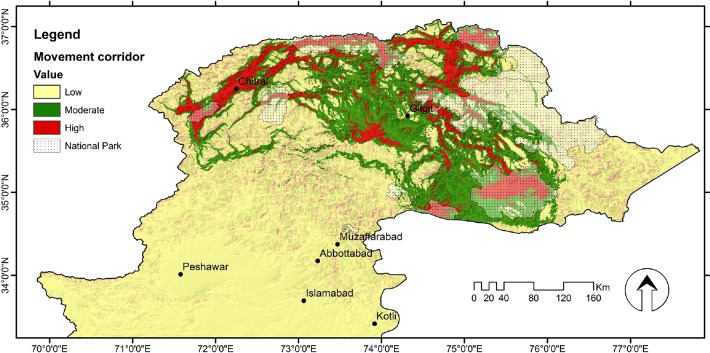
Potential movement corridors of wolf in northern Pakistan. Red areas are strong links while yellowish areas are weakest. Map also illustrating population connection found in National Park.

The corridor modelling generated estimates of habitat connectivity among scattered wolf populations in northern Pakistan (Fig 6). Four chunks of suitable habitat were identified within the Himalayas, Pamirs, Hindukush, and Karakorum mountains ranges of northern Pakistan. Sub-populations have strong, but unprotected connections and corridors movement existed between all major areas of wolf habitat. The model identified weak linkages between populations found at lower altitudes with high disturbance rates. Among the PAs, Chitral Gol, Broghil and Qurumber National Park had wide potential corridors comprised of suitable habitat connecting core areas. Furthermore, the analysis revealed appropriate dispersal habitat between Musk Deer and Deosai National Park, and similarly between Qurumber, Broghil, and Khunjerab National Park to allow for wolf dispersal.

## Discussion

### Maxent model and movement corridors of grey wolf in Pakistan

This study represents the first large-scale assessment of wolf distribution, habitat suitability and movement corridors in Pakistan. Our model identified areas with suitable habitat and corridors through which wolves may travel to reach new territories across northern Pakistan. The model showed considerable predictive performance, showing AUC value > 0.9 that may be placed among the highest in published models [5, 55, 68] and excellent values of the True Skill Statistic, corresponding to a very high predictive capacity [69–71].

Overall, our model found that the most suitable areas for wolves are located in mountainous regions where human disturbance is limited. Yet, it was also clear that wolves are relatively flexible in their use of habitat at the landscape scale. Along the altitudinal gradients, wolf presence was recorded ranging from a moist temperate zone in Musk Deer National Park up to the alpine zone in Khunjerab National Park. In general, wolves could potentially live in any area where human tolerance and prey populations are adequate to support viable numbers [72]. Wolves show different patterns of habitat selection based on time (year, season, time of day) and areas in which they were observed [73, 74]. Our data supported previous observations that wolves occur in various types of habitat and shows low habitat specificity and high levels of ecological resilience compared with other large carnivores [34, 72, 75].

Wolf habitat selection patterns at a fine scale appear to be influenced by complex interactions between habitat attributes and human disturbances [73, 76]. We found that wolf presence depended on the type of anthropogenic disturbance in the area. Most roads in the study area are unpaved with minimum disturbance and traffic pressure. We observed that wolves avoided main roads and tracks, but followed livestock tracks and small, unpaved roads with low disturbance. We suspect that wolves use roads for traveling, scent-marking, and territorial patrolling, but have also developed cryptic behavioral responses to roads, likely driven by the increased risks associated with human presence [77, 78]. Wolves are likely to select secondary gravel or unpaved roads for hunting due to the greater visibility and mobility [20]. The presence of road networks may drive wolves toward suitable habitat types. Wolf’s tolerance to human disturbances increased in suitable habitat types [79].

The results of our model suggested that the distance to road was an important predictor of wolf presence [76, 80, 81]. This finding suggested that wolves may seek to minimize the probability of encountering humans this [80, 81].The proximity to rivers was the second most important predictor. Riparian habitats provide wolves with increased opportunities to hunt wild prey and are also important in den selection [82, 83]. In our landscape shepherds prefer moving along streams and established temporary stay which may also attracts wolves to feed on livestock. Wolves were concentrated in lower areas due to snow-caused aggregation of prey during the winter season. Frozen rivers and lakes are often used by wolves to travel faster [84] [85]. Others environmental variables such as altitude, annual precipitation and land cover were among the variables that contributed least to the SDMs for *C*. *lupus* in northern Pakistan.

We found that summer huts, temporary settlements, and grazing pastures limited wolf distribution. Wolves exhibited some tolerance towards humans, enabling them to persist within a mosaic of human-altered and naturally occurring habitat. Wherever primary habitat is rare, wolves tend to be dispersed in meadows and rangelands, or in less-natural landscapes such as mixed-use agro ecosystems [86,87]. A similar relationship between the number of inhabitants of settlements and avoidance of close surroundings by wolves was observed in Poland [88]. Wolf populations in closer contact with human-active areas indicated tolerance to human activities [89].

Resistance modelling indicated the presence of habitat corridors for wolves in northern Pakistan. These corridors could link potential habitats and movement corridor between PAs. Habitat connectivity is not uniform in the Himalayas and population connectivity between the Pamirs and Himalayas range is very weak i.e. based on our corridor modelling analysis. Wolf populations in the Hindu Kush appear to be well connected with the population of the Pamirs and Karakorum’s, which is also enhanced by the establishment of PAs, including the Broghil, Qurumber and Khunjerab National Parks. PAs have become islands of habitat within a mosaic of agriculture and development, and although at a slower rate than non-PAs, anthropogenic activities persist even within the boundaries of PAs [90]. Chitral, Broghil and Qurumber were identified as areas of likely wolf activity based on habitat quality and connectivity to other patches of high-quality habitat. The wolf population in Deosai appears to be connected with the population of Central Karakorum National Park, but only weak connected with other potential habitat. The wolf populations at the Musk Deer and Khanbari study sites appear to be isolated.

Highly suitable habitat was also detected outside of PAs with minimum levels of anthropogenic activities. For large carnivores, sub-optimal habitats might serve as corridors linking habitats necessary for survival and reproduction and also to prevent inbreeding depression [91]. Grey wolves are able to travel through habitats considered poor in the search for an area to form a new pack [92]. “Pioneering” wolves have been known to disperse over large distances and settle in new habitats far from the nearest source population [34, 93]. The populations of the Hindu Kush, Pamirs and Karakorum appear to be connected through movement corridors, but these needs to be protected to facilitate safe use by dispersing wolves.

### Model constraints

There are two main limitation to our model. First, no prey availability estimates were available. Second, data was only collected during winter and wolf habitat selection patterns may vary between seasons. The dataset was influenced by the species’ patchy distribution, its rarity throughout the landscape, its seasonal surface occurrence, and its location (when active) on steep and rocky (often impassable) terrain [94]. Previous studies showed that wolf distribution at the landscape scale was influenced primarily by prey availability and human infrastructure [95]. Assuming prey biomass varies with habitat type, studies on carnivores demonstrate the potential for deriving accurate habitat and connectivity models [96, 97].

### Application of habitat modelling

With respect to species presence, the model predicted habitat suitability reasonably well. Among the four major chunks of high-quality habitat identified, one is protected, one is partially protected, and the rest are weakly protected. The most suitable habitat in the Hindu Kush range (Khanbari) lacks PAs and has poor connections with other populations. The eastern part of the Pamir range and southern Himalayas are partially connected with the western Karakorum and northern Himalayan populations, respectively. Our study provides a better idea of where wolves may disperse to in case numbers increase in the future, and help to identify priority areas for community engagement, management zones and proactive planning [98].

Habitat models developed in the current study will support wolf conservation in three ways. First, habitat maps provide a tool to identify suitable habitat and movement corridors and provided a guide map for investing limited conservation resources. Second, wildlife managers prioritize the establishment of more PAs covering suitable habitat and movement corridors to extend the PA network for the long-term survival of wolf populations in Pakistan. Third, it is a challenging issue in northern Pakistan to manage protected areas in stringent categories, due to heavy dependence of people on natural resources.
